# Effects of Shellac Self-Repairing and Carbonyl Iron Powder Microcapsules on the Properties of Dulux Waterborne Coatings on Wood

**DOI:** 10.3390/polym15092016

**Published:** 2023-04-24

**Authors:** Wenbo Li, Xiaoxing Yan

**Affiliations:** 1 Co-Innovation Center of Efficient Processing and Utilization of Forest Resources, Nanjing Forestry University, Nanjing 210037, China; 2 College of Furnishings and Industrial Design, Nanjing Forestry University, Nanjing 210037, China

**Keywords:** microencapsulation, bifunctional coating, preparation technology, coating performance

## Abstract

Magnetic carbonyl iron powder (CIP) microcapsules were created by in situ polymerization using melamine resin as the wall material and CIP as the core material. They were mixed with shellac self-repairing microcapsules to prepare dual-functional wood coatings, and the effect of different amounts of CIP microcapsules in the Dulux Waterborne primer on the performance of the primer was investigated. The findings demonstrated that the core-wall ratio had a significant impact on the characteristics of CIP microcapsules. The microcapsule coating rate reached 57.7% when the core-wall ratio was 0.65:1. The maximum reflection loss of CIP microcapsules with the core-wall ratio of 0.70:1 is −10.53 dB. When the addition amount of shellac self-repairing microcapsules is 4.2%, and the additional amount of CIP microcapsules with a core wall ratio of 0.65:1 and 0.70:1 is 3.0%, the coating color difference is the smallest. The number of microcapsules causes a noticeable drop in the coating’s gloss, and the amount of microcapsules causes a small negative change in the coating’s adherence. With an increase in the number of microcapsules, the coating’s hardness, impact resistance, and tensile resistance first rose and subsequently fell. When the content of CIP microcapsules with core-wall ratio of 0.65:1 and 0.70:1 was 9.0%, the hardness, elongation at break and repair rate of the coating reached the best performance. According to a comprehensive analysis, when the content of CIP microcapsules with core-wall ratio of 0.70:1 is 9.0%, the coating has good performance. At this time, the coating has a color difference of 1.83, a glossiness of 19.3, an adhesion of 2 H, a hardness of 3 H, an impact resistance of 17 kg·cm, and a repair rate of 33.3%. This provides a technical basis for the application of multifunctional coatings on wooden substrates.

## 1. Introduction

With the continuous development of science and technology, there is more and more electromagnetic radiation around people, and the harm caused by electromagnetic radiation is increasing. For this reason, people are increasingly pursuing original and environmentally friendly materials [1,2]. As a natural material, wood is widely used in architecture, furniture, interior decoration and other ways because of its green, renewable, diverse and beautiful texture and other characteristics [3,4,5]. Due to its long growth cycle and natural defects (shrinkage from drying and swelling with moisture, easy breakage, etc.), it is necessary to protect wood to make its use more sustainable [6]. The common method is to paint the wood surface to beautify and protect the wood. Water-based coatings are safe, non-toxic, wear-resistant, aging resistant, and easy to use. However, waterborne coatings cannot adapt to the deformation of wood caused by shrinkage when dry and later expansion, and their toughness is far lower than that of typical organic coatings. They are more prone to cracking after drying, reducing the service life of the wood [7,8].

Microcapsule technology aims to use natural or synthetic polymer compounds to form a continuous film (wall material) to wrap a material (core material), so that the chemical properties and functions of the core material are not affected and can be protected by the wall material. At present, a common method is to wrap repair materials and add them to the coating through microcapsule technology [9,10,11]. Yan et al. [12] successfully prepared microcapsules using urea-formaldehyde resin as the wall material to enhance the waterborne coating. The results showed that the coating with microcapsules had better aging resistance and higher self-repair ability compared with the coating without microcapsules, and the repair rate was about 20.0%. Yan et al. [13] prepared urea-formaldehyde/epoxy resin microcapsules by two-step in situ polymerization, and added the microcapsules to the water-based wood coatings. The results showed that when the concentration of microcapsules was 10.0% and the core-wall ratio was 0.83:1, the comprehensive performance of the water-based wood coatings was the best. By adding self-repairing microcapsules into water-based wood coatings, the defects of water-based coatings can be effectively addressed, and the repair effect of coatings after external damage can be increased.

Applying magnetic materials to wood can not only support the role of beautiful decoration, but also broaden the range of applications for wood [14,15]. As a common magnetic material, carbonyl iron powder (CIP) has the characteristics of large magnetic loss intensity and low price. Adding CIP directly into the coating will lead to uneven distribution of the CIP, affecting the coating performance, and because CIP is a black powder, it will cover the original texture of the wood, thus affecting the aesthetics [16,17]. These defects can be effectively improved by microencapsulating magnetic materials and adding them into the coating. Gan et al. [18] prepared magnetic wood materials by precipitation of magnetic CoFe_2_O_4_ nanoparticles and SiO_2_ on the surface of wood by a hydrothermal method, which gave the wood excellent magnetic and anti-ultraviolet properties, and enhanced its mechanical properties. Tang et al. [19] synthesized magnetic coatings on a wood surface using a layer-by-layer self-assembly method. The modified wood has magnetism, which is improved with an increase in the number of assembly layers. These studies show that endowing wood with magnetism can improve its characteristics; however, research on the preparation of dual-functional coatings prepared by mixing self-repairing materials and magnetic materials has not been widely reported.

In this paper, CIP microcapsules were prepared with melamine resin as wall material and CIP as core material. Shellac self-repairing microcapsules and CIP microcapsules were added to the water-based coating for modification. Through the optical and mechanical tests of the water-based coating with two kinds of functional microcapsules, the effects of the structure and morphology of the microcapsules and of different amounts of different types of microcapsules on the coating performance were studied. The results showed that the addition of microcapsules not only improved the optical and mechanical properties of the coating, but also gave the coating self-repairing and wave-absorbing effects. It can increase the service life and multi-functional application of water-based coatings.

## 2. Materials and Methods

### 2.1. Experimental Materials

In Table 1, the experimental materials are displayed. Fiberboard was used as the substrate, with dimensions of 100 mm × 100 mm × 5 mm (Shangpin Bense Smart Home Co., Ltd., Zaozhuang, China).

### 2.2. Microcapsule Preparation and Experimental Design

#### 2.2.1. Pretreatment of CIP Core Material

The pre-treatment of the core material can remove impurities from it. Firstly, the CIP was put into ethanol and stirred ultrasonically for ten minutes. Then, the CIP was sucked up by a magnet and separated from the ethanol, removing the impurities in the CIP. The resulting CIP was washed and dried with ethanol and deionized water for later use [20,21].

#### 2.2.2. Preparation of Shellac Microcapsules

According to the previous experimental results, melamine resin was selected as the wall material, shellac liquid and rosin liquid as the core material, and the core-wall ratio used to prepare the microcapsules was 0.8 to 1 [22,23]. First, 13.52 g of 37% formaldehyde solution, 6.0 g of melamine, and 30 mL of deionized water were weighed and poured into a beaker. Triethanolamine was used to adjust the pH of the solution to about 9. Then the beaker was put into a 70 °C constant-temperature water bath, and the rotation speed of the water bath was adjusted to 600 rpm for 30 min reaction to obtain a wall material solution. Separately, 0.15 g Span-20 and 0.15 g Tween-20 were weighed as emulsifiers and poured into a beaker. Ethanol solvent (78.9 mL) was added. After the mixture was stirred and fully dissolved, 4.4 g shellac solution and 4.4 g rosin solution were added. The temperature was adjusted to 60 °C, the stirring speed was adjusted to 600 rpm and the mixture was allowed to react for 60 min to obtain the core material lotion. The prepared wall prepolymer was slowly poured into the core material lotion at the stirring speed of 600 rpm, and then ultrasonically mixed with the lotion for 15 min. After the ultrasonic treatment was completed, the mixed lotion was poured into a beaker and put it into a temperature water bath. The pH of the lotion was adjusted to about 3.5–5.0 with citric acid monohydrate, and then the temperature water bath was raised to 60 °C for constant temperature reaction for 3 h. After the reaction was completed, the obtained lotion was stored for 3 days. After 3 days, it was filtered and washed with ethanol and deionized water many times. The obtained product was dried in an oven at 40 °C. The white powder obtained comprised shellac microcapsules.

#### 2.2.3. Preparation of CIP Microcapsules

The orthogonal experiment is a statistical method used for designing experiments, which can effectively reduce experimental errors, maximize experimental efficiency and accuracy, and save experimental costs and time. In the experiments described here, four factors and three levels of an L_9_ (3^4^) orthogonal experiment were used. Melamine resin was selected as the wall material of the microcapsules, and the melamine/CIP microcapsules were prepared with the core-wall ratio, reaction temperature, stirring rate and reaction time as the influencing factors. According to the design of the orthogonal experiments, the shape, yield and coating rate of microcapsules were analyzed to determine the largest influencing factors on the formation of the microcapsules and the best plan for preparing them. The preparation process of the microcapsules was further optimized by single-factor experiments aimed at the largest influencing factors. The orthogonal experimental arrangement is shown in Table 2, and the amount of experimental raw materials is shown in Table 3.

The orthogonal experiment 1–9 # microcapsule samples were created in accordance with Table 2. Preparation of microcapsule sample 1 # is described here as an example of the preparation process. First, 5 g of melamine, 10 g of 37.0% formaldehyde solution and 40 g of deionized water were weighed and mixed into a beaker. The beaker was placed in a thermostat water bath and agitated at 600 rpm. The pH of the mixture was gradually increased using triethanolamine to roughly 9.0, and the thermostat water bath was heated to 70 °C for 30 min. After the reaction, a transparent liquid was obtained for use. Separately, 2.58 g of treated CIP was weighed and put into 23.22 g of deionized water as core material. The stirring speed of the mechanical mixer was set to 300 rpm and stirring was continued for 10 min. Then the wall material prepolymer was slowly added to the core material, and 8% concentration citric acid solution was added to adjust the pH to about 3.0; the reaction was maintained at 20 °C for 30 min. After the reaction was completed, the mixture was put in a beaker for 1 day, and the product was then filtered. During the filtration process, distilled water was used for washing. After suction filtration, the obtained product was put into a 40 °C oven to dry for 24 h. The resulting powder was the microcapsule sample. The preparation process for the remaining samples 2–9 # followed the same procedures as for sample 1 # [24].

For the single factor experiments, the fixed reaction temperature was 60 °C, the reaction time was 1 h, and the stirring rate was 800 rpm. The core-wall ratio was taken as a variable, with values of 0.55:1, 0.60:1, 0.65:1, 0.70:1, 0.75:1, and 0.80:1, respectively. Six groups of single-factor experiments were carried out. The single factor experimental materials are shown in Table 4.

### 2.3. Primer Coating Preparation

According to previous experimental results, when the content of shellac self-healing microcapsules is 4.2%, the self-healing effect of the primer coating is the best. For preparation of the coating, the total weight of the fixed primer coating was 4.0 g, the coating thickness was 60 μm, the addition of shellac self-repairing microcapsules was fixed at 4.2%, and CIP microcapsules with core-wall ratios of 0.65:1 and 0.70:1 were added, respectively. The additional amount of CIP microcapsules with core-wall ratio of 0.65:1 was 3.0% (coating named 3–1), 6.0% (coating named 3–2), 9.0% (coating named 3–3), 12.0% (coating named 3–4), 15.0% (coating named 3–5) and 18.0% (coating named 3–6), respectively. The additional amount of CIP microcapsules with core-wall ratio of 0.70:1 was 3.0% (coating named 4–1), 6.0% (coating named 4–2), 9.0% (coating named 4–3), 12.0% (coating named 4–4), 15.0% (coating named 4–5) and 18.0% (coating named 4–6), respectively. After stirring evenly, the coating was ready for use. The microcapsule-enriched primer was coated on the fiberboard and the thickness was controlled at 25 μm by a film preparation device. After drying, the topcoat was evenly added with a thickness of 35 μm. The performance of the paint film applied on the substrate was tested [25,26].

The same method was adopted for different mixtures. The evenly mixed primer was poured into the mold, three samples of the same proportion were prepared, and then dried in a 60 °C oven for 24 h. The coating was taken out of the mold and used to test the performance of the paint film.

### 2.4. Testing and Characterization

#### 2.4.1. Microcharacterization Test of CIP Microcapsules

The morphology of CIP microcapsules was characterized by optical microscope (OM) Zeiss Axio Scope A1. A small amount of sample was taken out and evenly covered on the glass slide, and then the cover glass was covered. The prepared sample was placed on the observation table, and then the microscope was adjusted to the appropriate magnification for observation.

#### 2.4.2. Calculation of Coating Rate of CIP Microcapsules

The 1.0 g CIP microcapsules were weighed and poured into a mortar, and recorded as M_1_. Because the CIP microcapsules were soluble in dilute hydrochloric acid, while the melamine resin was insoluble in dilute hydrochloric acid, the CIP microcapsules were fully ground in a mortar and soaked in dilute hydrochloric acid for 24 h, so that the capsule core fully reacted with dilute hydrochloric acid. After the reaction was completed, the solution was filtered. After drying, the weight was recorded as M_2_, that is, the weight of the wall material in 1.0 g CIP microcapsule. M_1_-M_2_ is the mass of the core in 1.0 g CIP microcapsules, and the coating rate W_j_ can be calculated according to Formula (1).

(1) $$W_{j}=\frac{M_{1}-M_{2}}{M_{1}}\times 100\%$$

#### 2.4.3. Test Methods for Bifunctional Coatings

Using to the national standard GB/T11186.3-1989 [27], the coating color difference was tested by HP-2136 colorimeter. The specimen was placed under the colorimeter in one position, and the L_1_^*^, a_1_^*^, and b_1_^*^ values at this time were recorded. Then, another position was used, and the L_2_^*^, a_2_^*^, and b_2_^*^ values at this time were recorded. The color difference between the two points was expressed by ΔE and calculated according to Formula (2).

(2) $$\Delta E={\left[{\left(\Delta L^{*}\right)}^{2}+{\left(\Delta a^{*}\right)}^{2}+{\left(\Delta b^{*}\right)}^{2}\right]}^{\frac{1}{2}}\;$$

where ∆L =L_1_^*^ − L_2_^*^, ∆a=a_1_^*^ − a_2_^*^, ∆b=b_1_^*^ − b_2_^*^.

The gloss of the coating was evaluated using an HG60S gloss meter in accordance with GB/T 4893.6-2013 [28]. The coating hardness was evaluated using an QHQ-A coating scratch tester in accordance with GB/T 6739-2006 [29]. The coating adhesion was evaluated using HQG film scriber in accordance with GB/T 4893.4-2013 [30]. The impact strength of coating was evaluated using CJQ coating impact tester in accordance with GB/T 1732-1993 [31]. The tensile strength and self-repairing calculation parameters of coatings were measured by a universal tensile testing machine. The self-healing effect was calculated by Formula (3).

(3) $$\eta =\frac{E_{H}-E_{S}}{E_{I}-E_{S}}\times 100\%\;$$

where η refers to repair rate, E_H_ refers to the breaking elongation of the coating film after repair for 24 h, Es refers to the breaking elongation of the coating film after scratching, E_I_ refers the breaking elongation of the coating film without a scratch.

The coating surface was characterized by Quanta-450 SEM. Red ink, 75% ethanol aqueous solution, 70% detergent, and 15% NaCl solution were selected for cold liquid resistance testing of the coating. The reason for the selection is that 75% ethanol solution represents a neutral liquid, red ink represents a heavier color liquid, 70% detergent represents daily necessities, and 15% NaCl represents alkaline liquid. These four liquids basically cover the types of liquids that can be encountered in daily use of furniture. After the test, the damage condition of the test area was observed and the cold liquid resistance level was evaluated.

#### 2.4.4. Electromagnetic Parameter Testing of CIP Microcapsules

The Agilent E8363C vector network analyzer was designed to test the electromagnetic parameters of CIP microcapsules and the theoretical reflection loss (RL) of the microcapsules was calculate according to Formulas (4) and (5).

(4) $$Z_{\mathrm{in}}=Z_{0}\sqrt{\frac{\mu_{r}}{\varepsilon_{r}}}\tan h\left[j\frac{2\pi \mathrm{fd}}{c}\sqrt{\mu_{r}\varepsilon_{r}}\right]\;$$

(5) $$\mathrm{RL}=20\log\frac{Z_{\mathrm{in}}-Z_{0}}{Z_{\mathrm{in}}+Z_{0}}\;$$

where $Z_{\mathrm{in}}$ is the material impedance, $Z_{0}$ is the spatial free impedance, h, f, d, and c are magnetic field strength, frequency, material matching thickness, and speed of light, respectively, $\mu_{r}\;$ and $\varepsilon_{r}$ are the complex magnetic permeability and complex dielectric constant of the material, respectively.

All tests were repeated four times, with an error of no more than 5% each time.

## 3. Experimental Results and Discussion

### 3.1. Morphological Characterization of CIP Microcapsules by Orthogonal Experiment

**Figure 1:**
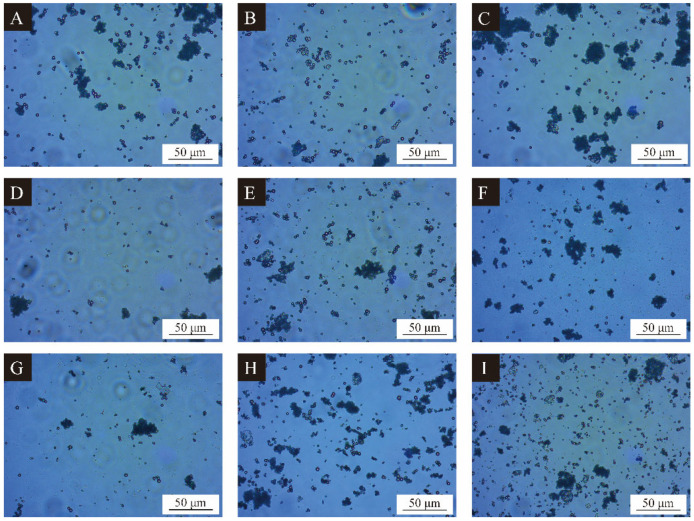
Optical microscope topography of orthogonal experimental products: (**A**) sample 1, (**B**) sample 2, (**C**) sample 3, (**D**) sample 4, (**E**) sample 5, (**F**) sample 6, (**G**) sample 7, (**H**) sample 8, (**I**) sample 9.

The morphology of microcapsules observed by OM is shown in Figure 1. It can be seen from Figure 1 that samples 1–9 # (Figure 1A–I) have a spherical particle shape; the particle edge is dark opaque spherical, and there are reflective highlights inside the particles. This is because the melamine resin is a continuous organism, which can be used as the shell structure of microcapsules, while CIP is a dispersion, which cannot be used as the shell structure of microcapsules. The spherical particles generated in the figure prove that melamine resin organism wraps around the CIP in a dispersion, forming a shell-core structure; the melamine resin successfully wraps CIP to obtain the microcapsules required for the experiment [32]. The particle size of sample 1 # (Figure 1A) microcapsule is about 6–8 μm, the shape is round, and there is a small amount of agglomeration. The reason may be that the stirring rate is too low, and the core material cannot be completely dispersed at low speed. The particle size of sample 2 # (Figure 1B) is uneven, about 2–9 μm. Some microcapsules are irregular spherical, and there is basically no agglomeration. The microcapsules of sample 3 # (Figure 1C) and sample 8 # (Figure 1H) are less formed and have serious agglomeration. The microcapsules of sample 4 # (Figure 1D) and sample 7 # (Figure 1G) are less formed, and the particle size is about 5–7 μm. The reason may be that the reaction temperature is too low, resulting in the incomplete reaction of melamine formaldehyde prepolymer and less wall materials. The microcapsules of sample 5 # (Figure 1E) and sample 6 # (Figure 1F) are relatively uniform in size, with a particle size of about 5–7 μm, and a small amount of aggregation. Sample 9 # (Figure 1I) microcapsule particle size is about 5–7 μm, and there is agglomeration. The reason may be that the core material is not completely covered due to the high core-wall ratio [33].

### 3.2. Analysis of Microcapsules Yield and Coating Rate

**Figure 2:**
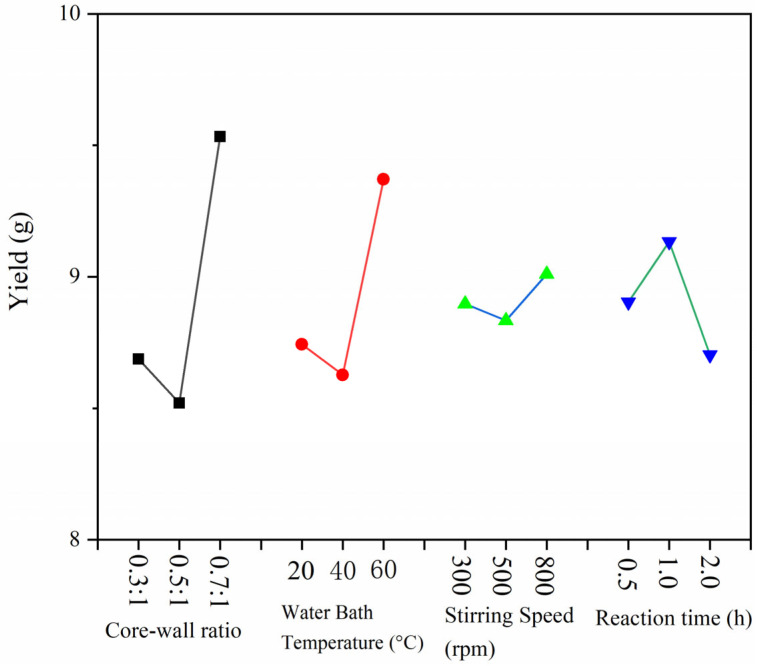
Yield range data of microcapsules in the test.

**Figure 3:**
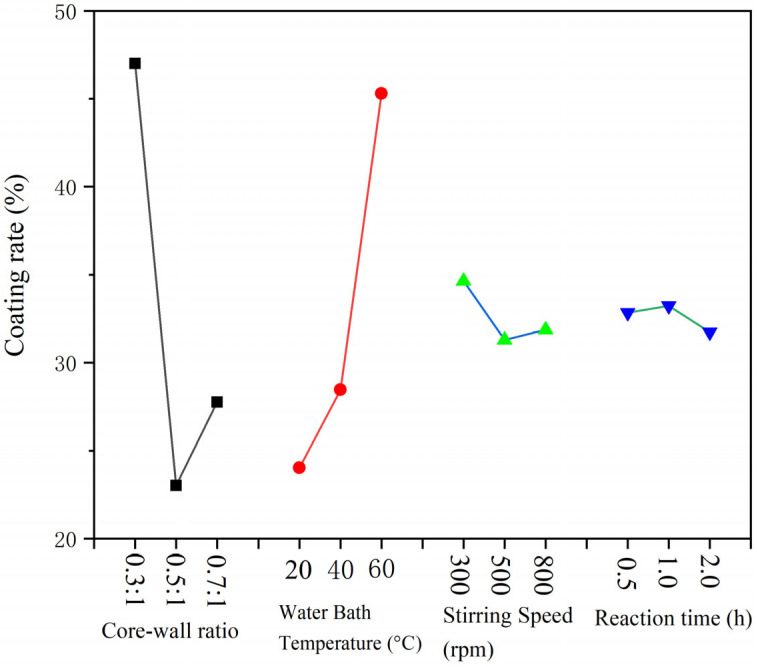
Coverage rate range data of microcapsules in the test.

In the orthogonal experiment, the yield range data from weighing 9 samples are displayed in Figure 2, and coating rate findings are displayed in Figure 3. It can be seen from Figure 2 that the core-wall ratio has the greatest impact on the production of microcapsules, while the other factors have only a small impact. The effect of stirring rate and reaction time on the production of microcapsules is relatively low. From the extremely poor results of the coating rate, it can be seen that the largest factor affecting the formation of microcapsules is the core-wall ratio, followed by the reaction temperature; the stirring rate and reaction time have little influence. According to the exploration of the early stage of the microencapsulation preparation process, as well as the factors of yield and coating rate, the following preparation method was found to be the best: 60 °C water bath temperature, 800 rpm stirring speed, and a 1.0 h reaction time. Next, the single-factor experiment was designed with the core-wall ratio as the variable.

### 3.3. Single Factor Experimental Analysis of Microcapsules

**Figure 4:**
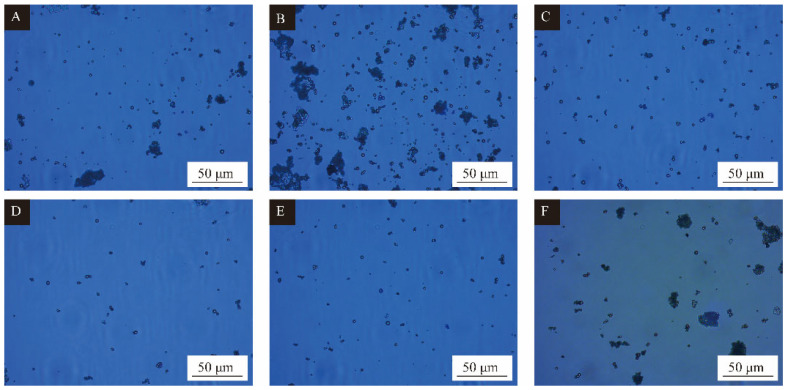
Single factor experimental microcapsule optical microscope topography: (**A**) core wall ratio 0.55:1, (**B**) core wall ratio 0.60:1, (**C**) core wall ratio 0.65:1, (**D**) core wall ratio 0.70:1, (**E**) core wall ratio 0.75:1, (**F**) core wall ratio 0.80:1.

**Figure 5:**
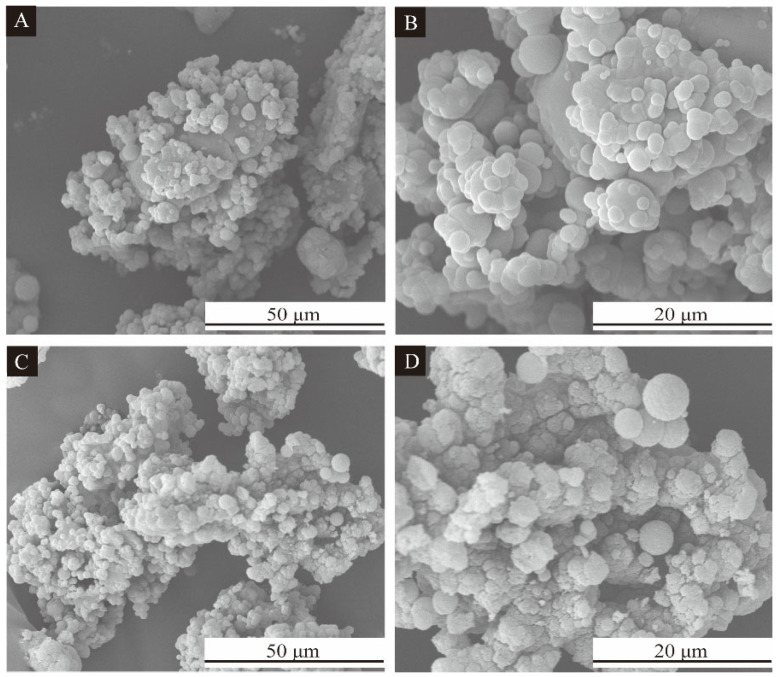
SEM morphology of single factor microcapsules: (**A**) low magnification of single factor 3′ # microcapsule, (**B**) high magnification of single factor 3′ # microcapsule, (**C**) low magnification of single factor 4′ # microcapsule, (**D**) high magnification of single factor 4′ # microcapsule.

**Figure 6:**
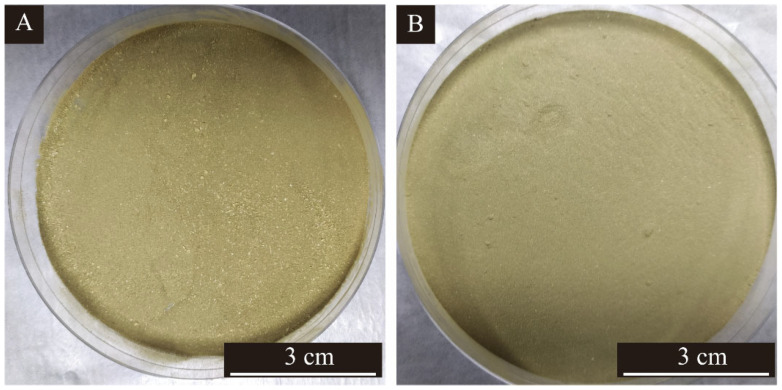
Macro photos of single factor microcapsules: (**A**) single factor 3′ # microcapsule, (**B**) single factor 4′ # microcapsule.

The morphology of microcapsules produced by single factor optimization with the core-wall ratio as a variable is shown in Figure 4, and the scanning electron microscope photos and macro photos of single factor samples 3′ # and 4′ # are shown in Figure 5 and Figure 6. Figure 4A shows a small amount of agglomeration, and the formation of microcapsules is less, with the particle size of about 1–6 μm. In Figure 4B, agglomeration is serious, and the particle size of the microcapsules is relatively large, about 8 μm. The microcapsules in Figure 4C,D have uniform particle size, about 6–7 μm, and almost no agglomeration. The microcapsules have a round appearance. In Figure 4E, some microcapsules are irregularly spherical with poor morphology and particle size of about 3–8 μm. Figure 4F shows aggregation and less microcapsules. The reason may be that too much microcapsule core material leads to uneven dispersion. According to this comprehensive analysis, the overall morphology and particle size of 3′ # and 4′ # microcapsules were better in the single-factor experiment. As can be seen from Figure 5, the surface of the microcapsule is smooth, round and spherical, with the best morphology.

### 3.4. Analysis of Single-Factor Microcapsules Yield and Coating Rate

**Figure 7:**
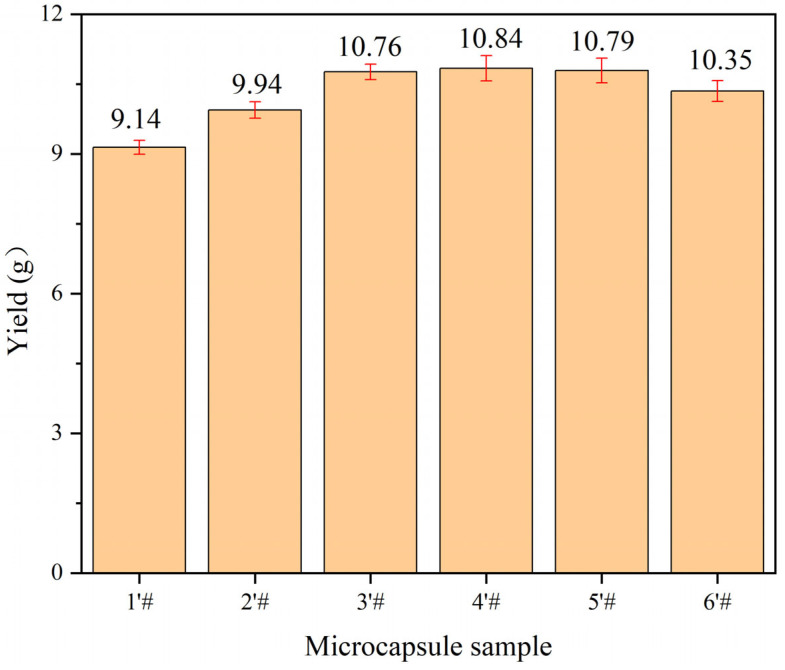
Single factor experimental microcapsule yield result.

**Figure 8:**
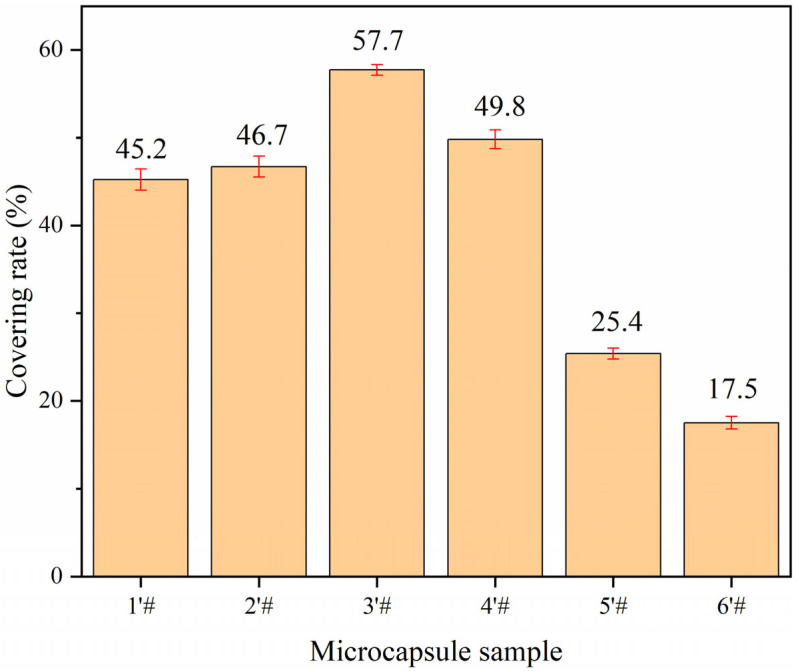
Single factor experimental microcapsule coverage result.

The evaluation results based on single-factor microcapsule yield and coating rate are shown in Figure 7 and Figure 8. When the quantity of core material delivery improved, the overall output of microcapsules first rose and subsequently fell. The reason is that with the increase of the core-wall ratio, the core material covered by the microcapsule wall material increases, and the resulting microcapsule quality increases. However, with the excessive delivery of the microcapsule core material, the core material cannot be completely dispersed in the solution, and the wall material cannot cover the core material well during the microcapsule preparation process, resulting in the reduction of the microcapsule output. The coating rate of microcapsules basically increased at first and then decreased with the increase of the core-wall ratio. When the core-wall ratio was increased from 0.55:1 to 0.65:1, the coating rate increased by 12.5%. When the core-wall ratio was increased from 0.65:1 to 0.80:1, the coating rate decreased by 40.2%. When the core-wall ratio was 0.65:1, the coating rate was the highest, reaching 57.7%. The initial increase of the core-wall ratio can enable the wall materials to cover more core materials. However, when the core material is too much, its dispersion becomes poor, and the level of core material agglomeration is serious, such that there is a decrease in the coating rate because the wall material cannot completely cover the core material.

### 3.5. Chemical Composition Analysis of Microcapsules

**Figure 9:**
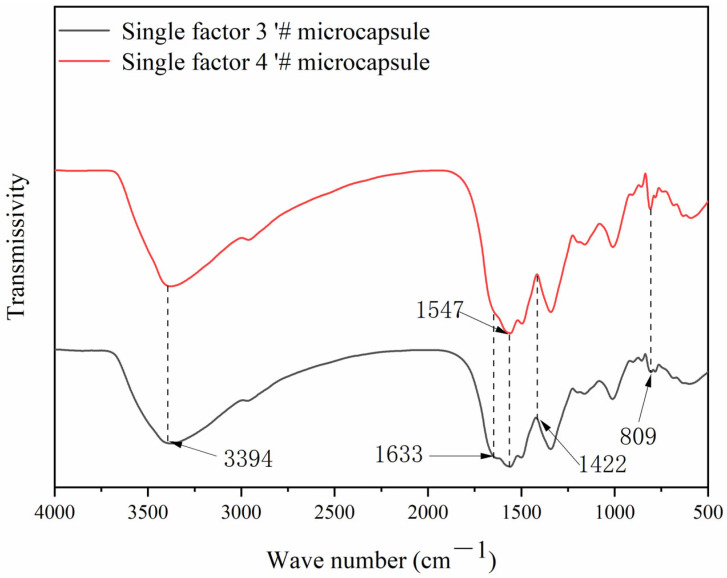
Infrared spectrogram of microcapsules.

Figure 9 shows the infrared spectra of single factor 3′ # and 4′ # microcapsules, from which it can be seen that the triazine ring bending vibration absorption peak appears at 809 cm^−1^, and the –NH– stretching vibration peak appears at 1547 cm^−1^, representing the characteristic peak of melamine resin, while the characteristic peak of CIP appears at 3394 cm^−1^ and 1444 cm^−1^, which indicates that the core material and the wall material have not undergone chemical reaction, and that the microcapsules have been successfully prepared [34,35].

### 3.6. Analysis of Electromagnetic Parameters and Microwave-Absorbing Properties of the Microcapsules

**Figure 10:**
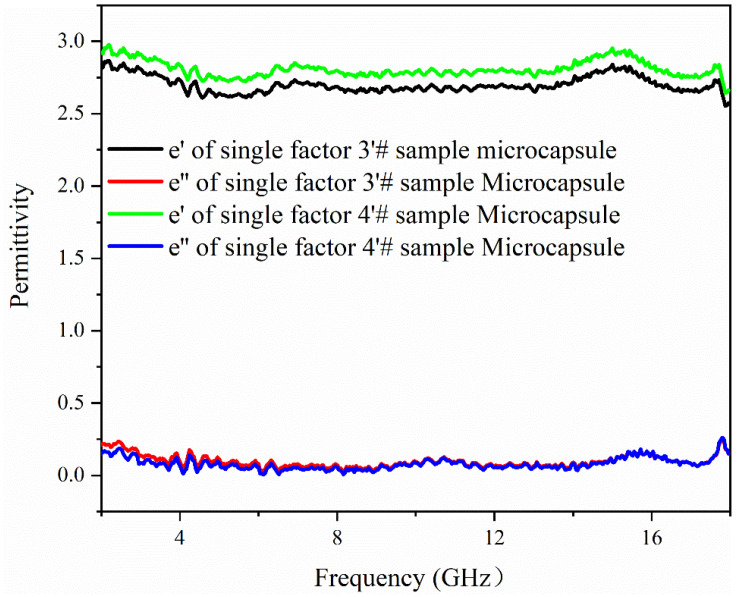
Permittivity of single factor 3′ # and 4′ # samples.

**Figure 11:**
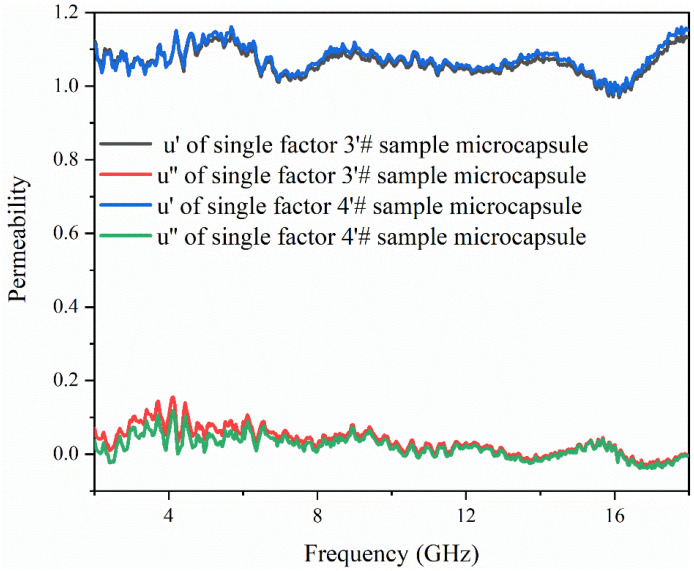
Permeability of single factor 3′ # and 4′ # samples.

**Figure 12:**
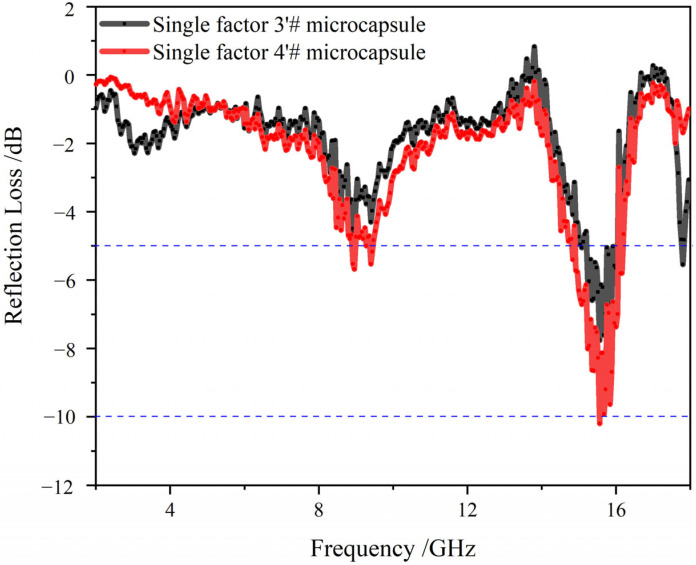
Theoretical reflection loss of single factor 3′ # and 4′ # samples.

Figure 10, Figure 11 and Figure 12 show the electromagnetic parameters and microwave-absorbing properties of single factor 3′ # and 4′ # microcapsules. As can be seen from the figure, the permittivity and permeability of the two different microcapsules are not much different, because although the core-wall ratio of the two microcapsules is different, they are made of the same absorbing material. When the matching thickness is 2.5 mm, the theoretical maximum RL of the single factor 4′ # microcapsule is −10.53 dB at 15.8 GHz, and the effective absorption bands are 8.9–9.7 GHz and 15.3–16.2 GHz, totaling 1.7 GHz. The test results show that the reflection loss is lower than that of D‘Aloia [36], and has met the user requirements.

### 3.7. Effect of CIP Microcapsules with Different Core-Wall Ratios on the Properties of Waterborne Wood Coatings

**Figure 13:**
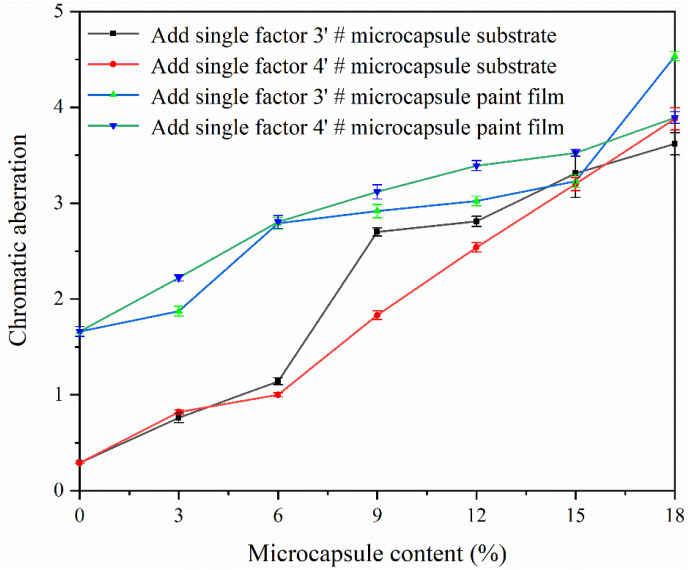
Effect of the different content of 3′ # and 4′ # microcapsules on color differences of paint film and of paint film on the substrate.

The color difference between the paint film applied on the substrate and the paint film is shown in Figure 13. The color difference of paint film and paint film on the substrate gradually increases with the increase of microcapsule content. This is because the CIP microcapsules are a gray-green powder; their own color affects the transparency of the coating, and when the content of microcapsules increases, the color difference is further increased due to uneven dispersion of microcapsules in the primer [37]. For the same added amount, there is little color difference from adding sample 3′ # CIP microcapsules and sample 4′ # CIP microcapsules. When the added amount of sample 3′ # and sample 4′ # microcapsules was 3.0–9.0%, the microcapsules had little effect on the color difference of the coating. The color difference of paint film is greater than that of paint film coated on substrate, because the color and gloss of the substrate will affect the color and appearance of the paint film.

**Figure 14:**
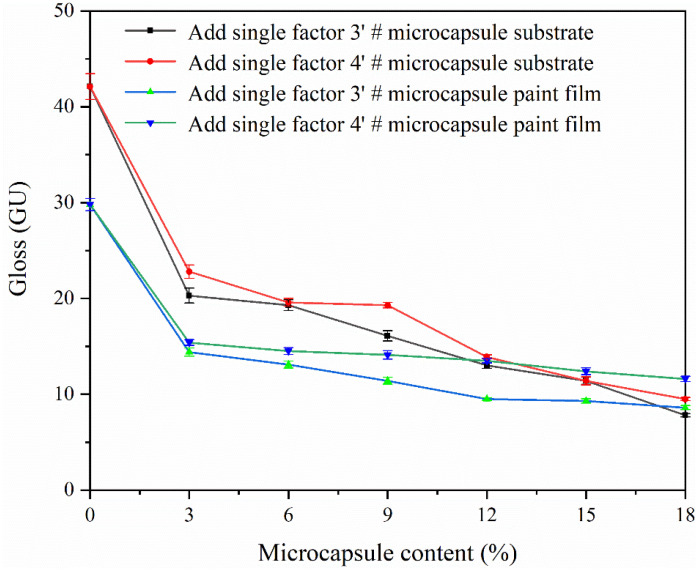
Effect of adding different contents of samples 3′ # and 4′ # microcapsules on the gloss of paint film and of paint film on the substrate under 60° glossiness.

The effect of different microcapsules and different microcapsule contents on the gloss of the coating is shown in Figure 14. The glossiness of paint film and of paint film on the substrate gradually decreased with the increase of microcapsule content. The reason for this phenomenon is that the CIP microcapsule is a spherical powder. The coating becomes rougher and rougher with the increase of the amount of microcapsules added. The microcapsule powder weakens the specular reflection ability of the coating, thus reducing the gloss of the coating [38]. When the content of CIP microcapsules is 3.0–9.0%, the effect on the gloss of the coating is relatively small. From the chart, it can be observed that the glossiness of the paint film without coating on the substrate is lower, and adding the single factor sample 4′ # CIP microcapsule coating has little effect on the gloss of the coating.

**Figure 15:**
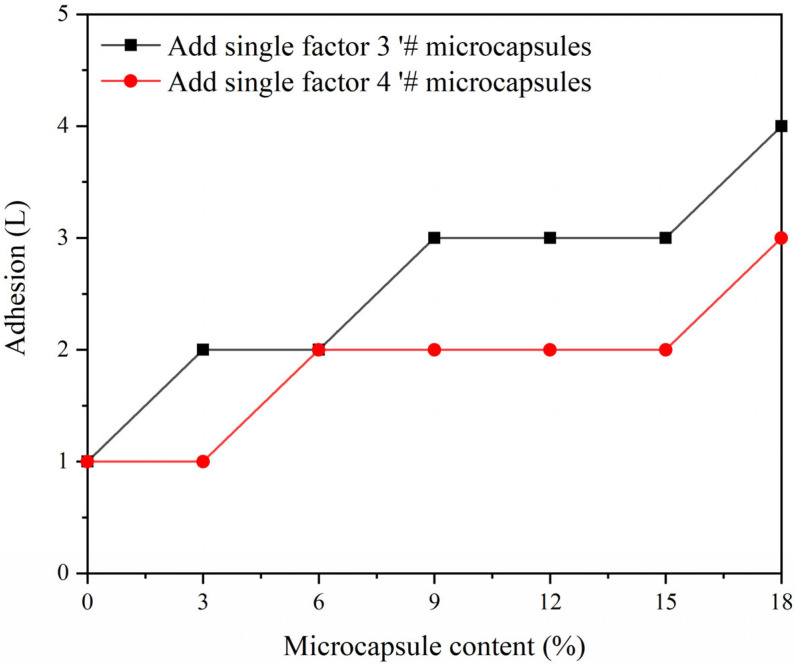
Effect of different content of 3′ # and 4′ # microcapsules on the adhesion of the coating.

**Figure 16:**
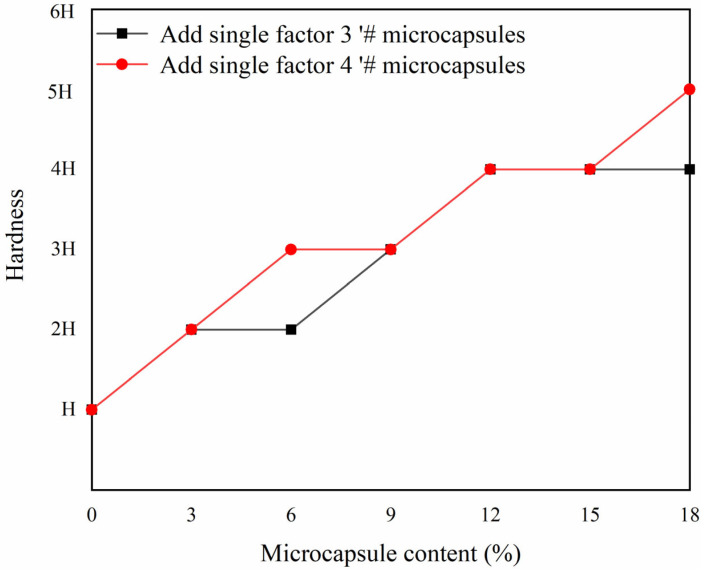
Effect of different content of 3′ # and 4′ # microcapsules on the hardness of the coating.

**Figure 17:**
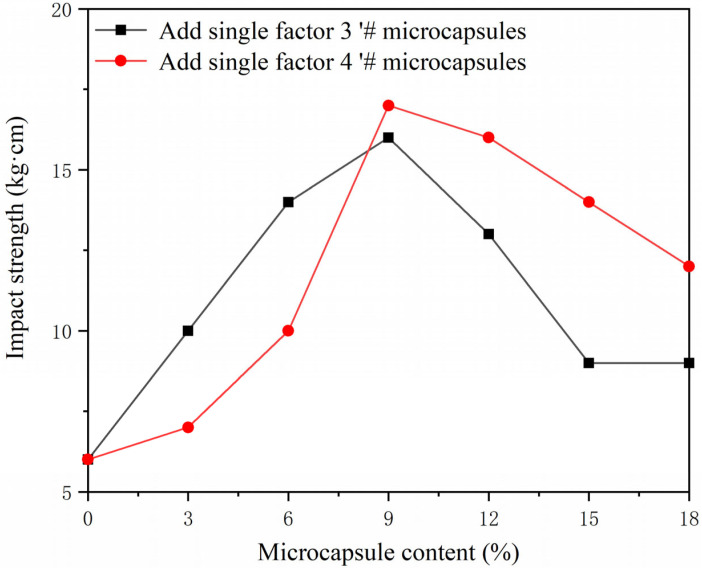
Effect of different content of 3′ # and 4′ # microcapsules on the impact strength of the coating.

The mechanical properties of the coating were tested as shown in Figure 15, Figure 16 and Figure 17. A blank sample was prepared for testing, to establish experimental reference lines and improve the accuracy of the experiment. The coating adhesion of single factor sample 3′ # microcapsules and single factor sample 4′ # microcapsules decreased gradually, but the overall effect on adhesion was not significant. This is because microcapsules are added to the primer. When the content of microcapsules is gradually increased, the particles in the coating increase, which affects the interface bonding ability of the coating and the wood substrate. It can be seen that the coating adhesion with single factor sample 4′ # microcapsules is relatively better. The overall hardness of the coating shows an upward trend. When the microcapsule content of sample 4 # was 18%, the hardness of the coating reached the maximum value of 5 H, which is 2 H higher than that of Yan et al. [13]. When the amount of microcapsules was increased, the impact strength of the coating first rose and then fell. The impact strength of the coating reached its maximum, which was 17 kg·cm, at 9% microcapsule concentration in sample 4′ #. This is because the wall material of the microcapsules, melamine resin, has good toughness [39]. Adding microcapsules to the water-based coating can improve the toughness of the coating, thus improving the impact resistance of the coating. However, when the amount of microcapsules is too high, it cannot be evenly dispersed in the water-based coating, and the agglomerated microcapsules reduce the impact resistance of the coating [40,41].

**Figure 18:**
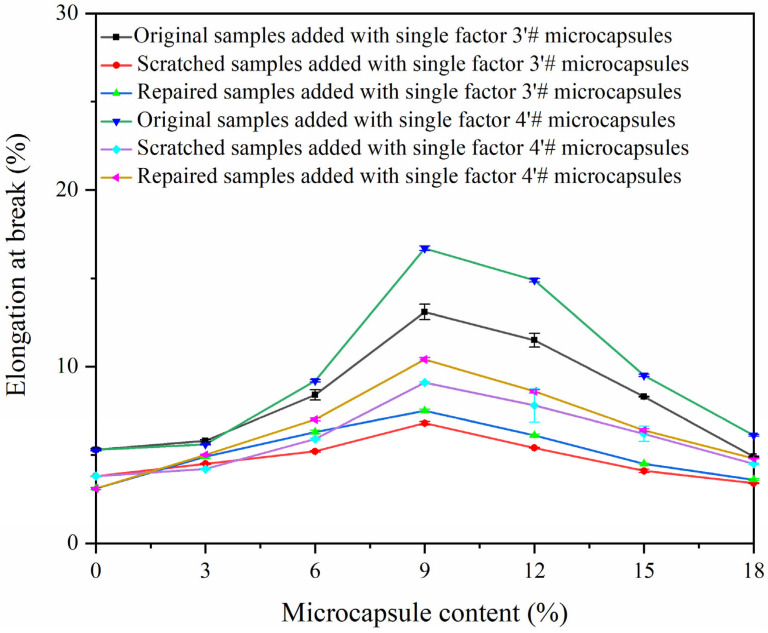
Effect of adding different contents of samples 3′ # and 4′ # CIP microcapsules on the elongation at break of the original coating.

A 38 × 19 mm single-sided blade (Shanghai Chengna Trading Co., Ltd., Shanghai, China) was used to draw cracks on the prepared paint film. The universal mechanical testing machine was used to carry out mechanical tensile tests on three modes of coating film (original coating film, coating film after scratch, and coating film after 24 h repair). The elongation at the break of the coating is shown in Table 5 and Figure 18. As the amount of microcapsules in the coating was increased, the breaking elongation initially increased and then gradually decreased. Melamine resin, as the wall material of the microcapsules, can increase the toughness of the coating, thus improving the elongation at break of the coating. However, when the content of microcapsules in the coating is too high and the powder in the coating increases, with uneven dispersion, the coating becomes brittle, and the toughness of the coating decreases, thus making the coating brittle and easily fractured [42,43]. When the content of sample 4′ # microcapsule was 9%, the elongation at coating break was the highest, reaching 16.7%. The repair rate of the coating was calculated according to Formula (3). Table 5 demonstrates that the coating without microcapsules had no repair effect, which may be due to the further expansion of the scratch of the coating under the influence of the environment due to the absence of the repair agent in the coating. The coating with microcapsules first increased and then decreased with the increase of the amount of microcapsules. When the content of CIP microcapsules is 3–6%, the shellac self-repairing microcapsules are broken by scratches, and the repair fluid flows out of the microcapsules to repair the coating. At the same time, the wall material of the CIP microcapsules, melamine resin, increases the toughness of the coating and prevents the coating from further expanding the scratches due to the influence of the external environment [44]. When the content of CIP microcapsules is 9–18%, because the shellac self-repairing microcapsules have quantitative limits, the coating toughness decreases as the powder in the coating increases. When 6% of sample 3′ # microcapsule was added, the coating repair rate was the highest, at 34.4%. The repair rate was increased by 13.8% when compared to Yan et al. [13].

**Figure 19:**
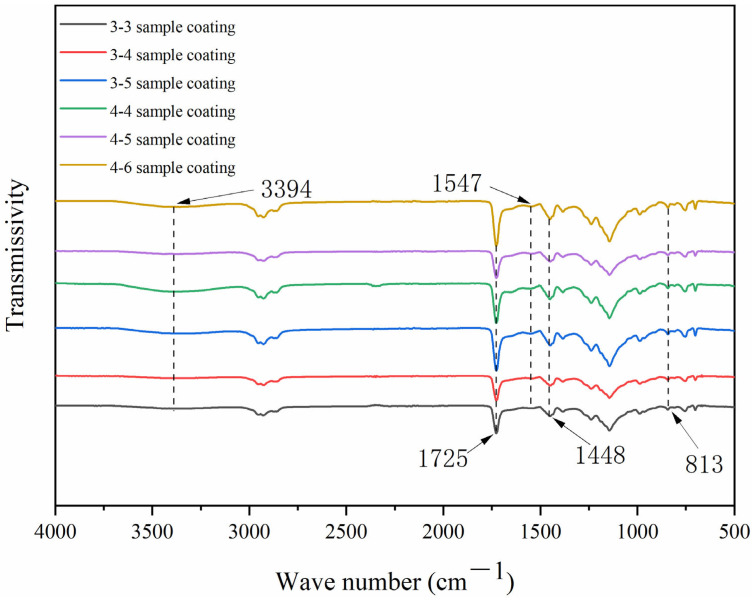
Infrared spectrogram of paint film.

Figure 19 shows the infrared spectrum of the coating before self-repair. It can be seen that there are characteristic peaks of CIP at 3394 cm^−1^ and 1448 cm^−1^, characteristic peaks of melamine resin at 813 cm^−1^ and 1547 cm^−1^, and characteristic peaks of shellac at 1725 cm^−1^. These peaks show that the addition of microcapsules has not changed the composition of the coating, and there is no chemical reaction between them.

**Figure 20:**
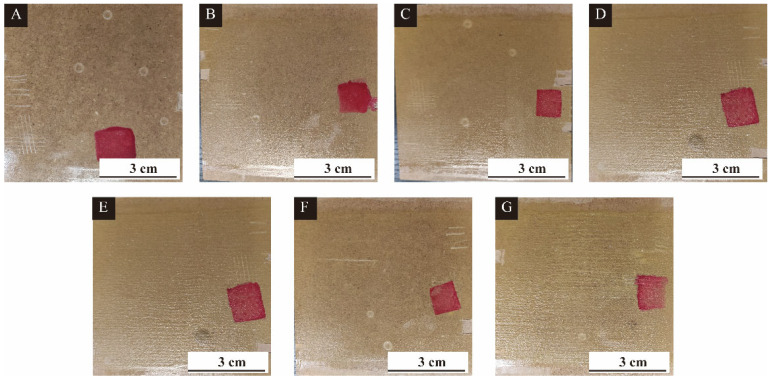
Macro photo of microcapsule coating with different contents of samples 3 # and 4 # added, respectively: (**A**) no microcapsule coating, (**B**) adding 6% 3′ # CIP microcapsules, (**C**) adding 9% 3′ # CIP microcapsules, (**D**) adding 12% 3′ # CIP microcapsules, (**E**) adding 3% 4′ # CIP microcapsules, (**F**) adding 6% 4′ # CIP microcapsules, (**G**) adding 9% 4′ # CIP microcapsules.

**Figure 21:**
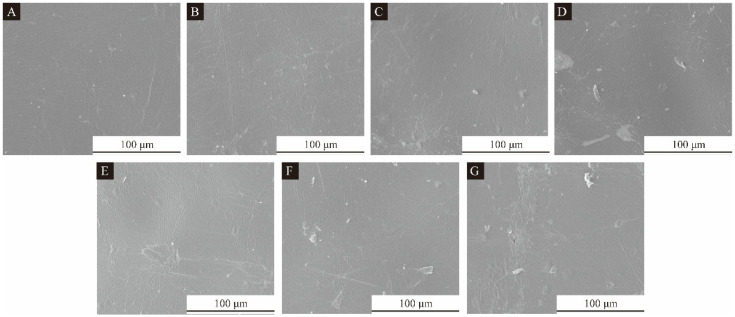
SEM morphology of microcapsule coating with different contents of samples 3 # and 4 # added, respectively: (**A**) no microcapsule coating, (**B**) adding 6% 3′ # CIP microcapsules, (**C**) adding 9% 3′ # CIP microcapsules, (**D**) adding 12% 3′ # CIP microcapsules, (**E**) adding 3% 4′ # CIP microcapsules, (**F**) adding 6% 4′ # CIP microcapsules, (**G**) adding 9% 4′ # CIP microcapsules.

Based on the above test results, with 9% content the performance of single factor sample 3′ # is the best, and with 6% content the performance of single factor sample 4′ # is the best. Figure 20 is a photo of the sample before and after liquid resistance. The red ink resistance is poor, with obvious traces. The resistance to 75% ethanol aqueous solution, 70% detergent, and 15% NaCl solution, respectively, is good, without any traces. Figure 21 shows the results of environmental scanning electron microscopy for conditions of no microcapsule coating, microcapsules added with single-factor sample 3′ # (6%, 9% and 12%) and microcapsules added with single-factor sample 4′ # (3%, 6% and 9%). The coating surface without adding CIP microcapsules is flat. When adding 6% content of single factor 3 # sample and 3% content of single factor 4 # sample, the coating has some small bulges, and the microcapsules are basically evenly dispersed in the coating. However, with the increase of the amount of microcapsules, the bulges in the coating become more obvious, since the increased amount of microcapsules cannot completely disperse in the water-based coating.

## 4. Conclusions

Through the four factor three level orthogonal experiment, it was found that the largest factor affecting the formation of CIP microcapsules was the core-wall ratio. When the core-wall ratio is 0.65:1, the morphology of the microcapsules is spherical, the particle size is about 6–7 μm and the coating rate is 57.7%. When the matching thickness is 2.5 mm, the theoretical maximum RL of the single factor 4′ # microcapsule is −10.53 dB at 15.8 GHz. When the CIP microcapsules with core-wall ratios of 0.65: 1 (3′ #) and 0.70: 1 (4′ #) were added to primer, and the addition of shellac self-repairing microcapsules was controlled at 4.2%, the lower the content of CIP microcapsules, the smaller the influence on color difference, with a minimum of 0.29. The glossiness and adhesion decreased with the increase in the amount of microcapsules. The hardness, impact resistance and tensile resistance of the coating first increased and then decreased with the increase in the amount of microcapsules. When the content of microcapsules with core-wall ratio of 0.65:1 was 9%, the hardness reached the best value at 3H, the elongation at break reached its best at 13.1%, and when the content was 6%, the repair rate was 33.4%. When the content of microcapsules with core-wall ratio of 0.70:1 was 9%, the hardness reached its best at 3H, the elongation at break reached its best at 16.7%, impact resistance reached its best at 17 kg·cm, and when the content was 6%, the repair rate was 33.3%. Comprehensive analysis shows that the coating performance is good when the addition of shellac microcapsules is 4.2%, the core-wall ratio is 0.70:1 and CIP microcapsule content is 9%. The research results show that the addition of shellac and CIP microcapsules improves the comprehensive performance of waterborne coatings, providing a technical reference for the application of multi-functional coatings on wood substrates, and broadening the application scenarios of waterborne coatings.

## Figures and Tables

**Table 1:** List of experimental materials.

Experimental Materials	Molecular Mass (g/mol)	CAS	Manufacturer
99.9% melamine	126.12	108-78-1	Nanjing Houxin Biotechnology Co., Ltd., Nanjing, China
37% formaldehyde	30.03	50-00-0	Jinan Chuangtong Chemical Co., Ltd., Jinan, China
triethanolamine	149.1882	102-71-6	Jinan Chuangtong Chemical Co., Ltd., Jinan, China
12.5% yellow shellac solution	-	-	Shanghai Yuhe Industrial Co., Ltd., Shanghai, China
rosin solution	-	-	Shenzhen Maner Technology Co., Ltd., Suzhou, China
Span-20	346.459	133-39-2	Nanjing Houxin Biotechnology Co., Ltd., Nanjing, China
Tween-20	604.813	9005-64-5	Nanjing Houxin Biotechnology Co., Ltd., Nanjing, China
CIP	195.897	13463-40-6	Nangong Xindun Alloy Welding Material Spraying Co., Ltd., Nangong, China
absolute ethanol	46.07	64-17-5	Wuxi Jingke Chemical Co., Ltd., Wuxi, China
citric acid monohydrate	210.14	5949-29-1	Nanjing Quanlong Biotechnology Co., Ltd., Nanjing, China
0.05 mol/L dilute hydrochloric acid	36.46	7647-01-0	Nanjing Kehua Test Reagent Consumables, Nanjing, China
Dulux waterborne primer	-	-	Akzo Nobel Paint (Shanghai) Co., Ltd., Shanghai, China

**Table 2:** Orthogonal test arrangement.

Sample	Core-Wall Ratio	Water Bath Temperature (°C)	Stirring Speed (rpm)	Reaction Time (h)
1 #	0.3:1	20	300	0.5
2 #	0.3:1	40	500	1.0
3 #	0.3:1	60	800	2.0
4 #	0.5:1	20	500	2.0
5 #	0.5:1	40	800	0.5
6 #	0.5:1	60	300	1.0
7 #	0.7:1	20	800	1.0
8 #	0.7:1	40	300	2.0
9 #	0.7:1	60	500	0.5

**Table 3:** Orthogonal experiment: raw material consumption.

Sample	CIP (g)	Deionized Water for Core (g)	Melamine (g)	37% Formaldehyde (g)	Deionized Water for Wall (g)
1 #	2.58	23.22	5.00	10.00	40.00
2 #	2.58	23.22	5.00	10.00	40.00
3 #	2.58	23.22	5.00	10.00	40.00
4 #	4.30	38.70	5.00	10.00	40.00
5 #	4.30	38.70	5.00	10.00	40.00
6 #	4.30	38.70	5.00	10.00	40.00
7 #	6.02	54.18	5.00	10.00	40.00
8 #	6.02	54.18	5.00	10.00	40.00
9 #	6.02	54.18	5.00	10.00	40.00

**Table 4:** Single factor experiments: raw material consumption.

Sample	Core-Wall Ratio	CIP (g)	Deionized Water for Core (g)	Melamine (g)	37% Formaldehyde (g)	Deionized Water for Wall (g)
1′ #	0.55:1	4.73	42.57	5.00	10.00	40.00
2′ #	0.60:1	5.16	46.44	5.00	10.00	40.00
3′ #	0.65:1	5.59	50.31	5.00	10.00	40.00
4′ #	0.70:1	6.02	54.18	5.00	10.00	40.00
5′ #	0.75:1	6.45	58.05	5.00	10.00	40.00
6′ #	0.80:1	6.88	61.92	5.00	10.00	40.00

**Table 5:** Elongation at break and repair rate of microencapsulated coating with different content of samples 3′ # and 4′ # CIP.

Sample	Addition Amount of Microcapsule (%)	Elongation at Break (%)			Repair Rate (%)
		Original Sample	Scratched Sample	Sample After Repair	
Blank sample	0	5.3 ± 0.2	3.8 ± 0.1	3.1±0.1	0
3–1	3	5.8 ± 0.2	4.5 ± 0.2	4.9 ± 0.2	30.8 ± 1.5
3–2	6	8.4 ± 0.4	5.2 ± 0.2	6.3 ± 0.3	34.4 ± 1.7
3–3	9	13.1 ± 0.6	6.8 ± 0.3	7.5 ± 0.3	11.1 ± 0.5
3–4	12	11.5 ± 0.5	5.4 ± 0.2	6.1 ± 0.3	11.5 ± 0.5
3–5	15	8.3 ± 0.4	4.1 ± 0.2	4.5 ± 0.2	9.5 ± 0.4
3–6	18	4.9 ± 0.2	3.4 ± 0.1	3.6 ± 0.1	1.3
4–1	3	5.6 ± 0.2	4.2 ± 0.2	4.6 ± 0.2	28.6 ± 1.4
4–2	6	9.2 ± 0.4	5.9 ± 0.2	7.0 ± 0.3	33.3 ± 1.6
4–3	9	16.7 ± 0.8	9.1 ± 0.4	10.4 ± 0.5	17.1 ± 0.8
4–4	12	14.9 ± 0.7	7.8 ± 0.3	8.6 ± 0.4	16.9 ± 0.8
4–5	15	9.5 ± 0.4	6.2 ± 0.3	6.4 ± 0.3	6.1 ± 0.3
4–6	18	6.1 ± 0.3	4.5 ± 0.2	4.6 ± 0.2	6.2 ± 0.3

## Data Availability

Not applicable.
